# Protective Factors in Resilient Volunteers Facing Compassion Fatigue

**DOI:** 10.3390/ijerph17051769

**Published:** 2020-03-09

**Authors:** Rosaura Gonzalez-Mendez, Matilde Díaz, Laura Aguilera, Julia Correderas, Yanira Jerez

**Affiliations:** Departamento de Psicología Cognitiva, Social y Organizacional, Universidad de La Laguna, 38200 La Laguna, Canary Islands, Spain; macadine@ull.edu.es (M.D.); laguiler@ull.edu.es (L.A.); juliacorrederasorozco@gmail.com (J.C.); yanira2b@gmail.com (Y.J.)

**Keywords:** volunteers, resilience, compassion fatigue, compassion satisfaction, happiness, well-being, post-traumatic growth

## Abstract

Volunteers may be exposed to the negative consequences of dealing with human suffering, such as compassion fatigue. However, very little is known about the protective factors that contribute to their resilience. The aim of this study was to analyze the extent to which different strengths (psychological endurance, purpose, and social support), orientations to happiness, and compassion satisfaction predict volunteers’ resilient outcomes (subjective well-being and post-traumatic growth) and compassion fatigue. Participants were 116 Spanish Red Cross volunteers (77.8% women). They were separately classified into three groups (low, medium, and high) according to the 33rd and 66th percentile scores on each resilient outcome. Univariate analyses of variance and post-hoc comparisons computed separately showed significant differences in most factors analyzed, except compassion fatigue. Logistic regressions revealed that endurance, organization support, and eudaimonia allowed for the correct classification of 83.3% of those high in post-traumatic growth (82.2% of the true-positives and 84.4% of the true-negatives). In addition to endurance and organization support, purpose was the strongest predictor of well-being (85.7% were correctly classified, 82.8% of the true-negatives and 88.2% of the true-positives). Finally, lower endurance predicted compassion fatigue (65.7% and 61.3% of the true-negatives and 69.4% of the true-positives). Findings indicate ways to promote resilience among volunteers.

## 1. Introduction

Volunteers play an essential role in different agencies and nonprofit organizations that work for the good of different sectors of the population, hence the interest in identifying the factors that make the management and promotion of volunteering possible [1,2,3]. Research has examined volunteer motivations and other factors (satisfaction, integration in the organization, perceived self-efficacy, etc.), since these are key in both the initiation and the duration of volunteer activity [4,5]. By contrast, the consequences of volunteering for those who do it have taken longer to arouse the interest of researchers, especially when these consequences are beneficial [2]. So far, very few studies have investigated resilience in volunteers. This makes it necessary to identify protective factors that contribute to resilience in this group. 

A large body of research has shown the negative effects faced by professionals working with distressed groups, thus pointing to the difficulties that volunteers might also encounter. Professionals who work with traumatized populations may share the emotional burden of the people they are trying to help [6,7,8,9]. This process may lead to unwanted changes in emotions, cognitions, and behaviors [10]. Researchers have used different terms to depict these changes [11,12], which have the potential to negatively affect professional exercise and well-being [13]. Vicarious trauma refers to harmful changes in professionals’ views of themselves, others, and the world, which take time to develop, whereas secondary traumatization is associated with a set of symptoms that resemble post-traumatic stress disorder and often have acute onset. Moreover, [14] introduced the term compassion fatigue to capture the negative effects of repeated exposure to details of people’s traumatic experiences. Defined as “the cost of caring” [15], it is the most commonly used term to refer to the consequences of secondary traumatic stress (e.g., hyperarousal, distressing emotions, functional impairment, etc.). It does not emanate from workplace stressors, but from direct relationships with suffering people [15]. 

Research has focused more recently on the benefits of helping for professionals. Those who have contact with the suffering of others may experience compassion fatigue and compassion satisfaction simultaneously [12,16]. Compassion satisfaction has also been associated with a lower risk of difficulties such as burnout [17]. Moreover, researchers have identified vicarious resilience among therapists as a result of contact with resilient people who have experienced trauma [18,19]. 

As with professionals, not only may volunteers be exposed to the negative consequences of dealing with human trauma, but their activity could also be an opportunity for satisfaction and growth. For instance, an increased sense of self-efficacy has been detected after participating in stressful events [3]. Moreover, [20] found that volunteers who had participated in potentially traumatic events showed high levels of resilience despite their experiences. In this study, resilience was primarily operationalized as the absence of symptoms of psychopathology, although measures of attachment, social support, and psychological quality of life were also included. Taking it a step further, we were interested in analyzing whether volunteers may also thrive thanks to their activity, as well as identifying strengths on which it is possible to intervene (i.e., non-static factors that may be targeted to promote resilience). 

Resilience has been defined in different ways, but most definitions refer to positive adaptation in the face of disturbing experiences [21]. This often means maintaining or recovering positive physical or mental health [22]. However, resilience may also lead to thriving when people experience positive changes that involve developing new coping skills, bolstering social relationships, or broadening perspectives [23,24,25]. Thriving after negative experiences connects the concepts of resilience and post-traumatic growth [26]. These two psychological constructs are different, yet empirically related [27]. While resilience is a broader category not necessarily linked to extreme events [23], post-traumatic growth is a possible response to traumatic experiences. The latter is therefore used as a resilient outcome together with other indicators of well-being and healthy functioning. 

A common distinction in well-being literature is between hedonic and eudaimonic well-being [28]. Hedonia is associated with the presence of pleasure and satisfaction of desires and is often operationalized as subjective well-being [29]. By contrast, eudaimonia extends well-being beyond pleasure, adopting a variety of interpretations, such as flourishing [30] or personal growth [31], among others. However, rather than being considered as indicators of subjective well-being and growth respectively, they are often characterized as different orientations to happiness. According to [32], orientations and behaviors reflect the choices a person makes, whereas experiences and functioning are often outcomes of those choices. Therefore, we considered well-being and post-traumatic growth as resilient outcomes in this study, and orientations to happiness as predictor variables. 

Moreover, we wanted to examine the association between resilient outcomes and certain non-static strengths with the potential to be useful targets for intervention. To select these strengths, we used the Resilience Portfolio Model [26]. According to this model, resilience is the process by which individuals, groups, or communities use different strengths and resources to achieve positive adaptation or thriving after adversity. Although both strengths and resources are considered protective factors, this model focusses on the former, which are grouped into three domains. Regulatory strengths are involved in controlling impulses, managing emotions, and persevering in the face of obstacles. Interpersonal strengths help get social support from different sources. Meaning-making strengths help finally make sense of life in coherence with adverse experiences. Although the model predicts that higher levels of density and diversity of strengths make resilience more likely, research has found that certain strengths, such as psychological endurance, purpose, or social support, are strong predictors of resilient outcomes [33]. In this regard, we selected these three strengths, each of which represents a different dimension to be analyzed in this study. 

In short, the aim of this study was to analyze the extent to which different strengths (psychological endurance, purpose, and two forms of social support), orientations to happiness (hedonia and eudaimonia), and compassion satisfaction predict volunteers’ resilient outcomes (subjective well-being and post-traumatic growth) and compassion fatigue. We expected that volunteers high in post-traumatic growth and subjective well-being would show higher scores on all factors than those classified as low or medium in each of the resilient outcomes. By contrast, we expected that those classified as high in both resilient outcomes would also show lower compassion fatigue.

## 2. Materials and Methods 

### 2.1. Study Design and Participants

A cross-sectional design was used to analyze the quantitative data from an online survey. Participants were 116 Spanish Red Cross volunteers (77.8% women) whose ages ranged from 20 to 84 (M = 40.6, SD = 11.85). They were all active at the time of the study and all lived in the same region of Spain. The participants had volunteered 6.5 years on average (SD = 6.8). The areas in which they volunteered were very diverse. Many collaborated in different social projects (27.4% collaborated in projects with the elderly, people with disabilities, gender-based violence survivors, children at risk, people in situations of extreme vulnerability, and immigrants). In addition, 22.2% worked in emergencies, 11.1% assisted in the search for employment, 5.1% worked with young people, and the rest volunteered in different areas, such as management, the environment, and International Cooperation.

### 2.2. Procedure and Ethical Considerations

The study received the approval of the Institutional Review Board of the Universidad de La Laguna (CEIBA2019-0357) and of the local leader of Spanish Red Cross (Tenerife, Spain). A person in charge of the organization gave volunteers’ phone numbers to researchers, but no other information that allowed us to identify them. The researchers explained the objectives of the study to each of the volunteers by telephone. The anonymity of their responses and the confidentiality of the data were guaranteed at all times, stressing the independency of the research team with respect to the organization. Once they had agreed to participate (only 1.7% declined), they received a link to an online survey. Their informed consent was obtained in writing before the survey responses were collected. Any information that allows participants to be identified, such as gender, has been removed from the database.

### 2.3. Measures

In addition to some general questions (age, years or months dedicated to volunteering, area in which they collaborated, etc.), the survey included different scales aimed at assessing compassion satisfaction and compassion fatigue, orientations to happiness, strengths related to resilience, and resilient outcomes. 

**Compassion satisfaction and compassion fatigue.** Two subscales of the Spanish version of the Professional Quality of Life Scale (ProQOL) [34] were used to assess compassion satisfaction and compassion fatigue. The third subscale, burnout, was discarded for this study due to its low internal consistency. The 10-item subscale of compassion satisfaction assesses satisfaction with one’s ability as an effective caregiver (e.g., ‘I like to work helping people’). The subscale of compassion fatigue also consists of 10 items, which reflect the negative consequences derived from the traumas of the people with whom one works (e.g., ‘I find it hard to separate my personal life from my professional life’; ‘I feel like I am the one who experiences the trauma of someone I have helped’). Response options for all the subscales were (0 = *never*, 1 = *rarely*, 2 = *a few times*, 3 = *somewhat often*, 4 = *often*, and 5 = *very often*). Internal consistency was calculated using Cronbach’s alpha, with values of 0.86 and 0.78, respectively. 

**Orientations to Happiness.** We used the Spanish short version [35] of the Orientations to Happiness Questionnaire [36]. This version consists of six items. Three referred to the Hedonic component (‘life is too short to postpone the pleasures it can provide’; ‘in choosing what to do, I always take into account whether it will be pleasurable’; ‘for me, the good life is the pleasurable life’). The other three items assess the Eudaimonic orientation (‘my life serves a higher purpose’; ‘I have a responsibility to make the world a better place’; ‘what I do is important to society’). Response options ranged from 1 (*very much unlike me*) to 5 (*very much like me*). Cronbach’s alpha reached values of 0.79 and 0.70, respectively. 

**Strengths associated with resilience.** We chose two scales from the Resilience Portfolio Measurement Packet [37] to measure two strengths (Purpose and Psychological Endurance) that have been found to predict resilient outcomes [33,38]. Specifically, we used two items to assess purpose (e.g., ‘my life has a clear sense of purpose’; ‘I have a good sense of what makes my life meaningful’) and an eight-item scale to measure psychological endurance (‘people rely on me through good times and bad’; ‘I believe that what doesn’t kill you makes you stronger’). In both cases, response options ranged from 4 (*mostly true*) to 1 (*not true*). Cronbach’s alpha were 0.83 and 0.78, respectively.

Moreover, we also used a scale developed by [39] to assess two forms of social support. It consists of five items that measure informal social support (e.g., ‘I feel that I have the unconditional support of some people around me’; ‘I can rely on my family and / or friends when I have difficulties’) and two items that evaluate formal social support. In this latter case, we adapted the items to ask about the support received from the organization where they volunteered (‘I have had the help of various professionals of this organization’ and ‘whenever I had a problem I felt supported by people from the organization’). Response options of this scale ranged from 0 (*does not describe me at all*) to 3 (*fully describes me*). Cronbach’s alpha were 0.82 and 0.76, respectively. 

**Resilience outcomes.** We assessed both post-traumatic growth and subjective well-being. For measuring post-traumatic growth, we used a nine-item scale from the Resilience Portfolio Measurement Packet [37]. This scale measures increased strengths, spiritual change, new life possibilities, etc. (e.g., ‘I changed my priorities about what is important in life’; ‘now I know that I can handle hard times’). 

Also from the Resilience Portfolio Measurement Packet [34], we used a 13-item scale to measure subjective well-being (e.g., ‘I am satisfied with my life’, ‘I have a lot to be proud of’). In this case, Cronbach’s alpha was 0.90. Response options of the two scales ranged from 4 (*mostly true*) to 1 (*not true*). Cronbach’s alpha were 0.80 and 0.90, for each of them.

### 2.4. Statistical Analysis

Data analysis was performed in several stages, using SPSS Statistics 22 (IBM, Armonk, NY, USA). First, all scale scores were standardized by converting them to z-scores, thus allowing us to compare scales with different response ranges. Z-scores also facilitate the calculation of probabilities. Descriptive analyses were also computed. Second, to better understand the differences between thriving and lower levels of functioning, we compared the participants classified as “low” (those who scored below the 33rd percentile), “medium” (between the 33rd and the 66th percentile), and “high” (higher than the 66th percentile) in each resilient outcome (post-traumatic growth and well-being). After testing the homogeneity of variances, using Levene’s test, univariate analyses of variance (ANOVAs) and post-hoc comparisons were carried out separately for each of these two outcomes. Welch’s test was used when variances were not homogeneous. 

After confirming the linear relationships between each of the outcomes and the other variables, we selected the two more extreme groups (low and high) to better identify the strengths that contribute to above-average resilience outcomes. Then, we computed two binary logistic regressions to predict post-traumatic growth and subjective well-being, separately. Finally, we computed a new binary logistic regression to predict compassion fatigue.

## 3. Results

### 3.1. Zero-Order Correlations

Table 1 shows zero-order correlations between the factors analyzed. Post-traumatic growth and well-being were positively associated with all factors analyzed, except compassion fatigue, which did not show any significant correlation. Post-traumatic growth also did not show an association with hedonic happiness, but it did with eudaimonic happiness. Orientations to happiness did not show an association with any strength. Age only positively correlated with years dedicated to volunteering (*r* = 0.20, *p* < 0.05). By contrast, the years spent volunteering were positively related to the support that participants considered receiving from the organization (*r* = 0.29, *p* < 0.01) and negatively related to eudaimonic orientation to happiness (*r* = −0.24, *p* < 0.01). 

### 3.2. Contrast between Different Levels of Post-traumatic Growth and Subjective Well-being

ANOVA comparing volunteers with different levels of post-traumatic growth showed significant differences in all factors analyzed, except compassion fatigue and hedonic orientation to happiness (Table 2). As expected, post-hoc analyses confirmed that participants high in post-traumatic growth showed higher scores on most factors than those classified as low or medium, with the sole exception of hedonia. By contrast, the results failed to support the differences hypothesized in compassion fatigue.

ANOVA comparing volunteers with different levels of subjective well-being also showed significant differences in most factors analyzed, except compassion fatigue and the two orientations to happiness (Table 3).

### 3.3. Prediction of Resilient Outcomes

As shown in Table 4, psychological endurance, social support from the organization, and eudaimonic orientation to happiness predict higher levels of post-traumatic growth. This model allowed for the correct classification of 83.3% of the participants (84.4% of the true-negatives and 82.2% of the true-positives). 

The model to predict subjective well-being also included psychological endurance and organization support as significant predictor variables (Table 5). However, purpose appears as a specific strong predictor in this case. This model correctly classified 85.7% of the participants (82.8% of the true-negatives and 88.2% of the true-positives). 

### 3.4. Prediction of Compassion Fatigue

Finally, the model computed to predict compassion fatigue revealed that lower psychological endurance increased the risk of this problem (*B* = –0.52, *SE* = 0.26, Wald = 3.97, *df* = 1, *p* < 0.05, odds ratio [*OR*] = 0.59, 95% confidence interval [*CI*] = [0.36, 0.99]). This model allowed for the correct classification of 65.7% of the participants (61.3% of the true-negatives and 69.4% of the true-positives).

## 4. Discussion

This study was aimed at analyzing the extent to which different strengths (psychological endurance, purpose, and two forms of social support), orientations to happiness (hedonia and eudaimonia), and compassion satisfaction predict volunteers’ resilient outcomes (subjective well-being and post-traumatic growth) and compassion fatigue. 

Post-traumatic growth and subjective well-being are among the most commonly measured resilient outcomes. However, although they correlate with each other, they are still different constructs [40]. Consistently, we did find that both resilient outcomes correlated to each other, but they also showed some different results. Specifically, the main difference detected between the two measures was related to the orientations to happiness. While none of these orientations predicted subjective well-being, eudaimonia did increase the likelihood of post-traumatic growth. These results support the idea that hedonia and eudaimonia reflect different dimensions of happiness. Not only was the correlation between the two factors moderate, but they also showed different results in predicting post-traumatic growth. Eudaimonic orientation reflects people’s search for meaning and growth [28], which is compatible with successfully facing the difficulties of volunteering. By contrast, hedonic orientation was only related to subjective well-being. The orientations to happiness reflect the choices people make [32]. Hence, the results they obtain are in accordance with their choices. 

As expected, the results confirmed that those participants classified as high in both post-traumatic growth and well-being showed higher scores on most predictor variables than those classified as low or medium. In addition, endurance and organization support predicted both resilient outcomes, which is consistent with previous studies [33]. Thus, while endurance has proven useful to maintain effort in the face of adversity, getting support ensures that one will be able to cope better. Moreover, the results again highlight that the two resilient outcomes may show different patterns [40]. While well-being was specifically predicted by purpose, post-traumatic growth was predicted by eudaimonia. Having a purpose helps one mobilize one’s own resources to achieve personal objectives, but it seems to also help one feel good during the process. This may be because purpose is one of the meaning-making strengths that make resilience more likely [26], and dimensions of meaning have proven relevant to well-being at different ages [41]. In contrast with purpose, eudaimonia refers to the values that drive one to care for others, which seems to help one go on. Although in this study both outcomes were related, post-traumatic growth is not always associated with well-being [40], since resilience is not an all or nothing phenomenon [42]. People can show thriving even when they are struggling with difficulties and keep moving forward [43]. 

Moreover, no significant differences were found in compassion fatigue when comparing the volunteers with different levels of either post-traumatic growth or well-being. Therefore, the results did not support our expectation that those classified as high in both resilient outcomes would also show lower compassion fatigue. However, they are consistent with previous findings that indicate marginal symptom load in this type of sample [20]. As suggested by these researchers, the findings could be pointing out that volunteers are reluctant to recognize symptoms of distress due to their activity. However, it is also possible that the voluntary nature of their activity leads only those who feel more satisfied to remain in the organization. For instance, those with insecure attachment orientations can shorten volunteering due to a greater propensity for compassion fatigue or less engagement in volunteer activities [44,45]. It could also be argued that similar exposition to suffering necessarily leads to similar levels of compassion fatigue. However, it seems unlikely in this study, since the participants volunteered in different areas. A common exposure to suffering people could also account for similar levels of compassion fatigue. However, it seems unlikely, since the participants volunteered in different areas. 

Despite having not found significant differences between the groups of volunteers in compassion fatigue, the results did show that lower psychological endurance slightly increased the risk of this problem appearing. This again indicates that psychological endurance is a strength required in the face of difficult situations and that a lower score in this predictor places volunteers at a greater risk of feeling overwhelmed by circumstances. 

### Limitations

This study has some limitations that need to be considered. Its cross-sectional design does not allow us to establish any causal direction of the relationships analyzed. Therefore, it is not possible to know if resilient outcomes are a result of volunteering. Also, the sample size was not large enough to allow us to generalize the findings. In addition, volunteers were collaborating in areas that allegedly require different levels of emotional involvement. The researchers who conducted the study were independent and they had no connection to the organization. However, we cannot rule out a social desirability bias regarding compassion fatigue.

## 5. Conclusions

So far, very few studies have examined volunteers’ resilience and no one has analyzed thriving in the face of volunteer activity. In addition, the current study points to some non-static strengths that may be useful to promote resilience in volunteers. Based on the Resilience Portfolio Model [23], we selected some strengths to be analyzed. This model offers a theoretical framework that may help us understand these and other potential strengths for resilience in volunteers. 

The findings indicate that organization support and psychological endurance are significant predictors of both resilient outcomes, whereas well-being is linked to purpose and post-traumatic growth to eudaimonia. The improvement of organization support is apparently the easiest for aid organizations to handle. However, maintaining volunteer satisfaction and well-being requires integrating support with the assignment of tasks and increased responsibilities. In this regard, it is necessary to help volunteers find a purpose linked to the organization. In addition, aid organizations could benefit from knowing both volunteers’ motivations and their orientations to happiness before planning how to promote psychological endurance. While eudaimonic orientation refers to the values that drive one to care for others, purpose is rather related to personal goals. Since eudaimonic orientation seems not to be enough to maintain the commitment to the organization, it is necessary that aid organizations know volunteers’ personal purpose and promote its compatibility with the aims of the organization. According to Radey and Figley [16], distress produced by volunteering can be used to flourish, increasing affect, resources, and self-care to provide an optimal environment that leads to higher satisfaction. This implies continued attention to the well-being of volunteers, who often demand training, to be heard and valued, less bureaucratic tasks, and more resources and time to carry out their work.

## Figures and Tables

**Table 1:** Zero-order correlations between the factors analyzed.

Factors	1	2	3	4	5	6	7	8	9
1.Compassion satisfaction									
2.Compassion fatigue	−0.166								
3.Eudaimonic orientation	0.229 ^*^	0.147							
4.Hedonic orientation	0.188 ^*^	0.140	0.235 ^*^						
5.Endurance	0.510 ^**^	−0.149	0.173	0.122					
6.Purpose	0.405 ^**^	−0.109	0.139	0.147	0.627 ^**^				
7.Informal social support	0.294 ^**^	−0.070	0.126	0.112	0.472 ^**^	0.419 ^**^			
8.Organization support	0.353 ^**^	−0.016	0.111	0.053	0.232 ^*^	0.173	0.173		
9.Subjective well-being	0.408 ^**^	−0.035	0.239 ^**^	0.243^**^	0.665 ^**^	0.636 ^**^	0.473 ^**^	0.340 ^**^	
10.Post-traumatic growth	0.423 ^**^	0.002	0.291 ^**^	0.162	0.610 ^**^	0.400^**^	0.341^**^	0.353^**^	0.463 ^**^

* *p* < 0.05; ** *p* < 0.01.

**Table 2:** ANOVA for contrasting volunteers with different levels of post-traumatic growth.

Factors	Low (L)		Medium (M)		High (H)			Post-hoc			
	*Mean*	*SD*	*Mean*	*SD*	*Mean*	*SD*	*F*(2,113)	L-M	L-H	M-H	ղp^2^
Endurance	−0.69	0.94	0.06	0.67	0.66	0.73	31.73 ***	−0.75 **	−1.35 ***	−0.60 **	0.38
Purpose	−0.53	0.98	0.31	0.96	0.35	0.81	12.52 ***	−0.84 **	−0.88 **	-	0.18
Informal SS	−0.40	1.08	0.53	0.90	0.37	0.82	7.14 ^w^**	-	−0.77 **	-	0.12
Organization support	−0.35	0.92	−0.15	1.01	0.44	0.92	8.44 **		−0.79 ***	−0.59 *	0.13
Eudaimonia	−0.37	0.94	0.08	0.91	0.32	1.00	5.98 **	-	−0.69 **	-	0.10
Hedonia	−0.29	0.87	0.27	0.88	0.14	1.12	3.49 *	-	-	-	-
Compassion satisfaction	−0.42	0.99	−0.17	0.98	0.52	0.79	12.37 ***	-	−0.93***	−0.68 *	0.18
Compassion fatigue	−0.05	0.91	0.20	1.18	-0.06	0.98	0.68	-	-	-	-

^W^ Welch’s test. *** *p* ≤ 0.001. ** *p* ≤ 0.01. * *p* ≤ 0.05. (L) Low, (M) Medium, (H) High.

**Table 3:** ANOVA for contrasting volunteers with different levels of well-being.

Factors	Low (L)		Medium (M)		High (H)			Post-hoc			
	*Mean*	*SD*	*Mean*	*SD*	*Mean*	*SD*	*F*(2,113)	L-M	L-H	M-H	ղp^2^
Endurance	−0.87	0.90	0.00	0.89	0.74	0.56	36.82 ^w^***	−0.87 ***	−1.61***	−0.74 ***	0.35
Purpose	−0.87	0.90	0.00	0.84	0.73	0.67	30.37 ***	−0.87 ***	−1.60 ***	−0.73 ***	0.35
Informal SS	−0.53	1.09	−0.04	0.95	0.51	0.72	5.68 ^w^**	-	−1.05 ***	−0.55 *	0.15
Organization support	−0.43	0.98	−0.04	0.87	0.43	1.05	6.54 **	-	−0.86 **	-	0.10
Eudaimonia	−0.35	0.97	0.12	0.93	0.10	1.08	2.42	-	-	-	-
Hedonia	−0.25	0.94	−0.06	1.04	0.31	0.94	2.72	-	-	-	-
Compassion satisfaction	−0.60	1.02	0.41	0.90	0.45	0.90	10.15 ***	−0.64 *	−1.05 ***	-	0.15
Compassion fatigue	0.23	1.03	−0.08	0.91	−0.07	1.11	1.00	-	-	-	-

^W^ Welch’s test. *** *p* ≤ 0.001. ** *p* ≤ 0.01. *p ≤ 0.05. (L) Low, (M) Medium, (H) High.

**Table 4:** Results of the binary logistic regression to predict post-traumatic growth.

Predictors	*B*	Wald’s Test	*df*	*SE*	Odds Ratio	*p*	95% *CI*
Endurance	1.80	18.95	1	0.41	6.07	0.000	[2.69, 13.67]
Organization support	0.95	7.31	1	0.35	2.59	0.007	[1.30, 5.17]
Eudaimonia	0.70	4.86	1	0.32	2.02	0.028	[1.08, 3.76]

*B* = Regression coefficients. *SE* = standard error. *CI* = confidence interval.

**Table 5:** Results of the binary logistic regression to predict subjective well-being.

Predictors	*B*	Wald’s Test	*df*	*SE*	Odds Ratio	*p*	95% *CI*
Endurance	1.77	7.89	1	0.63	5.90	0.005	[1.71, 20.34]
Purpose	1.83	6.99	1	0.69	6.22	0.008	[1.60, 24.08]
Organization support	1.26	3.94	1	0.63	3.52	0.047	[1.02, 12.23]

*B* = Regression coefficients. *SE* = standard error. *CI* = confidence interval.
